# Editorial: Approaches for Severe and Persistent Mental Illness

**DOI:** 10.3389/fpsyt.2021.722496

**Published:** 2021-07-19

**Authors:** Anna Lisa Westermair, Scott A. Irwin, Ulrich Schweiger, Manuel Trachsel

**Affiliations:** ^1^Institute of Biomedical Ethics and History of Medicine, University of Zurich (UZH), Zurich, Switzerland; ^2^Clinical Ethics Unit, University Hospital Basel (USB) and University Psychiatric Clinics (UPK), Basel, Switzerland; ^3^Cedars Sinai Medical Center, Los Angeles, CA, United States; ^4^Department of Psychiatry and Psychotherapy, University of Lubeck, Lubeck, Germany

**Keywords:** severe and persistent mental illness, quality of life, palliative care, psychiatry, compulsory interventions, medical assistance in dying, ethics

Some persons diagnosed with severe mental disorders do not achieve sufficient symptom reduction and remain experiencing impaired psychosocial functioning and quality of life despite long-term, intense, and evidence-based treatment efforts by their healthcare team, extensive engagement by their relatives, and by themselves as patients. Those patients are considered “at risk of therapeutic neglect and/or overly aggressive care within current paradigms” (1).

For this patient population, the term *severe and persistent mental illness* (SPMI) has been coined, a concept which was analyzed by  Zumstein and Riese in the present Special Topic (2020). Reviewing the scientific literature on SPMI, the authors concluded that—although a consensus definition is lacking—the definitions of SPMI proposed to date commonly comprise the following three dimensions: diagnosis, duration, and disability. Diagnosis often includes schizophrenia, bipolar disorder, major depression, anorexia nervosa, and personality disorders. Duration refers to the duration of symptoms, treatment, or disability with disability referring to poor psychosocial functioning or quality of life.

The present Special Topic is concerned with new approaches to the multitude of practical as well as ethical challenges that mental health professionals are confronted with when caring for SPMI patients, for example regarding compulsory interventions. In a survey of psychiatrists in Switzerland by Stoll, Hodel et al., nearly all respondents found it important or very important to respect the autonomy of SPMI patients. Nonetheless, when confronted with case vignettes of severe, longstanding, therapy-refractory anorexia nervosa, schizophrenia, and major depression, approximately one in three of the same psychiatrists reported they would use compulsory interventions although patients were explicitly described as having preserved decision-making capacity despite their SPMI. This inconsistency speaks to the ethical conflict between respecting a patient's autonomy and benefiting the patient by disorder-modifying treatment.

Common in the context of SPMI, this conflict is also at the heart of the question of medical assistance in dying (MAiD) in mental disorders. Stoll, Ryan et al. conducted an ethical analysis of the role of perceived burdensomeness in requests for MAiD by SPMI patients. They concede that the perception of burdensomeness may be heavily influenced by the mental disorder, indicating a lack of decision-making capacity. However, perceived burdensomeness may also be a realistic assessment and part of the unbearable existential suffering motivating a request for MAiD. Therefore, Stoll, Ryan et al. on the one hand propose that SPMI patients should not be routinely excluded from MAiD. On the other hand however, patients with a lack of decision-making capacity should be protected from getting access to MAiD by additional safeguards.

In another article of the present Special Topic, Noppes et al. bring together the issues of compulsory interventions and MAiD in a case report of a woman with organic delusional disorder. Using compulsory admission and concealed administration of an antipsychotic, the patient's decision-making capacity could be restored which enabled her to enact a previously made plan of requesting assisted dying.

As a potential middle ground between compulsory interventions (valuing the ethical principle of beneficence over the principle of respect for patients' autonomy) and MAiD (valuing the ethical principle of respect for patients' autonomy over the ethical principle of beneficence), the adaption of palliative care for psychiatry has been proposed (1–4). While palliative care emerged as care for people dying of cancer, its scope has expanded considerably in the last decades. Palliative care is now believed to be indicated for a variety of diseases including heart failure (5), kidney disease (6), and neurodegenerative diseases such as dementia. The state of the art of palliative care in advanced dementia has been reviewed by Eisenmann et al. in the present Special Topic. The authors conclude that caring for persons with dementia poses specific challenges for which palliative care teams might not be prepared: the deterioration of cognitive functions such as reasoning, and communication impedes decision making as well as reliable assessment of symptoms. Also, behavioral symptoms may prove too disruptive on a palliative care ward as illustrated by the case report from Noppes et al. Here, the compulsory admission to a psychiatric inpatient setting was initiated when treatment on the palliative care ward could not be continued due to lack of cooperation caused by delusions and disordered thought.

While there is consensus that palliative care is indicated for persons with progressive organic psychiatric disorders such as dementia, palliative approaches for other mental disorders remain controversial. This controversy may be founded in an overly narrow conceptualization of palliative care as end-of-life care (7). However, while palliative care emerged as end-of-life care, it is not limited to it; rather, it is now understood as care for people with “serious health-related suffering” in general and should be offered depending on patient needs, goals, and priorities—not depending on prognosis (8). The primary goals of palliative care are relieving suffering and improving quality of life by means other than curing (or modifying) the illness (8). Adapting this modern understanding of palliative care to psychiatry, Palliative Psychiatry accepts that for some SPMI patients, curative (or disease-modifying) goals of care may become secondary to the relief of suffering (1, 2, 7). Supporting this proposition, four out of five psychiatrists in Switzerland found palliative care approaches indicated for certain persons with SPMI (9). In the present Special Topic, Decorte et al. detail a palliative care concept for persons with SPMI in the residential setting, called “Oyster Care.” The Oyster Care Team forms a “shell” that protects and supports the patients, enabling them to function as best as possible. The primary goal of Oyster Care is relief of suffering more than psychiatry's traditional goals of symptom reduction and improvement of the patients' ability to function on their own. Furthermore, adapting the holistic approach of Palliative Psychiatry (1, 2), Oyster care enriches the standard biopsychosocial model of understanding and treating mental disorders by the existential dimension. According to Decorte et al., Oyster Caregivers should have existential and spiritual competences not only to help patients identify and undertake meaningful activities, but also to cope with own existential distress such as feelings of powerlessness which frequently arises in the context of SPMI.

In sum, the present Special Topic provides a conceptual article on the notion of SPMI (Zumstein and Riese), and an overview of different approaches for SPMI including a discussion of compulsory interventions, the concept of Palliative Psychiatry and a particular form of it, Oyster Care. Furthermore, the possibility of MAiD for persons with SPMI is critically discussed.

With this Special Topic, we hope to enrich and stimulate the further scientific elaboration and research and the clinical implementation of promising approaches for SPMI.

## Author Contributions

AW wrote the first draft of this manuscript and MT critically revised it. All authors read and approved the final version.

## Conflict of Interest

The authors declare that the research was conducted in the absence of any commercial or financial relationships that could be construed as a potential conflict of interest.
